# On Collaborative Governance: Building Consensus on Priorities to Manage Invasive Species Through Collective Action

**DOI:** 10.1002/pei3.70029

**Published:** 2025-03-27

**Authors:** Shannon Colleen Lynch

**Affiliations:** ^1^ Department of Plant Pathology University of California Davis Davis California USA

**Keywords:** biological invasions, collaborative governance, consensus‐based, *Euwallaceae*, Fusarium dieback, invasive forest pests, stakeholder engagement

## Abstract

Invasive forest pests can spread across large landscapes that include numerous land‐use management jurisdictions. As such, forest pest invasions need to be addressed with collaborative efforts because a single entity is seldom able to respond to the full scope of the problem. A collaborative governance framework provides a collective decision‐making process that allows diverse sets of actors who share an interest in a policy or management issue to work together towards mutually beneficial outcomes. Here, I apply a theoretical model of collaborative governance to understand the conditions in cooperative decision‐making that led to a consensus on statewide priorities to control an important invasive pest in California, the Fusarium dieback invasive shothole borers (FD–ISHB) beetle‐pathogen invasion. This provides (1) an empirical case study of collaborative governance in action throughout the stakeholder consensus building process and (2) interrogates that case study for theoretical contributions to the literature on collaborative governance, with special focus on invasive species management. Cohesive process outcomes were born out of effective principled engagement, participants' deep understanding and dedication to the system context, and the salient forces of leadership and interdependence baked in throughout the project. Ultimately, participants devoted their time and energy to a short but intensive planning process, resulting in more capacity for joint action, trust, interdependence, and a robust action plan that was quickly implemented.

## 1 Introduction

The greatest opportunity to effectively manage biological invasions is at early stages. However, this timing means that urgent, costly, and politically difficult decisions must usually be made when there is still insufficient data about which areas are most vulnerable to an infestation, how the invaders spread across a complex landscape, how severe their impacts might be, and what management approaches are most effective among a variety of land‐use jurisdictions (Rotherham and Lambert 2012; Epanchin‐Niell et al. 2014; Epanchin‐Niell and Wilen 2015). As such, important and intertwined ecological and social considerations make effective action difficult. The ecological complexity of the invasion problems also broadens the social context to involve a wider variety of people who have a stake in the outcomes of management decisions (Bodin 2017; Crowley, Hinchliffe, and McDonald 2017). This scenario can be fodder for controversy and social disagreements, posing further challenges to invasive species management (Rotherham and Lambert 2012; Estévez et al. 2015; Crowley, Hinchliffe, and McDonald 2017). Moreover, conflicts might escalate or deescalate depending on the characteristics of the introduced species itself (e.g., life history features, charismatic qualities, and economic benefit) and the people, agencies, and institutions involved (Rotherham and Lambert 2012; Estévez et al. 2015; Crowley, Hinchliffe, and McDonald 2017).

The emergent tree pest‐pathogen complex Fusarium dieback–invasive shothole borers (FD–ISHB; Mendel et al. 2012; Eskalen et al. 2013) is an important and ongoing biological invasion in Southern California that involves a diversity of stakeholders, encompassing avocado production and urban‐wildland forest systems that confer essential economic benefits and ecosystem services. Indeed, the California avocado industry produces 90% of the United States domestic crop. Urban forests in California remove 567,748 t CO_2_ annually, equivalent to the annual output of 120,000 cars (McPherson, van Doorn, and de Goede 2016). Additionally, affected riparian forests in California are critical breeding habitat for endangered bird species, help filter pollutants, regenerate groundwater, and enhance recycling of nutrients (Ballard et al. 2004). The spread of the introduced beetles and fungi they carry and the impacts of the FD–ISHB invasion across these varied and complex landscapes have led to management challenges of great concern for different entities. FD–ISHB has resulted in the loss of hundreds of thousands of trees in riparian ecosystems of Southern California (Boland 2016; Parks 2017), and the avocado industry and cities have already spent over $5.5 million to combat the pest‐pathogen complex (Parks 2017). For urban forests, initial projections suggest that FD–ISHB has the potential to kill roughly 27 million trees (38%) in Southern California's 10,992‐km^2^ urban region (McPherson, van Doorn, and de Goede 2016). Crossing agricultural, wildland, and urban ecosystems under private, municipal, state, and federal control means that the FD–ISHB issue is beyond the ability of any single organization to address the full scope of these devastating impacts on the environment, public health, and economic vitality of diverse social‐ecological systems.

Given that invasive pests such as FD–ISHB are characterized by their ability to move across dynamic geographic and social boundaries, a collective action process involving stakeholder groups, policymakers, and researchers is required to address the problem. In contrast to top‐down regulatory and technocratic solutions that have proven successful in protecting individual species or solving “end of the pipe” pollution problems (e.g., Clean Air Act of 1970, Clean Water Act of 1972), a collaborative governance strategy is often necessary to manage transboundary issues such as source pollution, climate change, and biodiversity protection (Gerlak, Heikkila, and Lubell 2012). Indeed, “command‐and‐control” forms of regulation governing environmental resources face demands by citizens, businesses, and non‐profit organizations for more participatory processes and access to public decision making (Ebrahim 2004; Holling and Meffe 1996).

Moreover, transboundary issues—such as those associated with forest pest invasions—need collaborative efforts because one single entity is seldom able to address the full scope of the problem (Bryson, Crosby, and Stone 2006; Emerson, Nabatchi, and Balogh 2012). Collaborative governance is part of a worldwide trend pushing towards greater decentralization of environmental governance and is defined as “… a collective decision‐making process that allows diverse sets of actors who share an interest or stake in a policy or management issue to work together toward mutually beneficial outcomes” (Gerlak, Heikkila, and Lubell 2012). This kind of decision‐making is particularly applicable to “common pool resources” such as fisheries, forests, and water (Wade 1987; Ostrom 1990; Sigurdson, Stuart, and Bratty 2011).

The essential building blocks for integrative pest management (IPM) to control FD–ISHB was initiated and informed by informal collaborative governance arrangements with the California Avocado Commission, The Nature Conservancy, the Natural Communities Coalition of Orange County, Irvine Ranch Conservancy, OC Parks, and San Diego Association of Governments. These initial arrangements among a collection of industry, governmental, and non‐governmental actors evolved into a formal statewide collaborative action effort through new legislation to confront the problem. In 2018, the California Legislature passed, and Governor Brown approved Assembly Bill No. 2470 (A.B. 2018) which authorized the California Invasive Species Council (CISAC) to build a consensus plan “…for the cure or suppression of diseases associated with the spread of Invasive shothole Borers, including, but not limited to the Polyphagous and Kuroshio shothole borers” and allocated $5 million to execute the plan. The CISAC committee directed the development of the plan that addressed four key elements and corresponding sub‐committees: (1) greenwaste and firewood as pathways; (2) research and technology development; (3) survey, detection, and rapid response; and (4) outreach and education (Table 2).

CISAC's efforts meet the criteria of collaborative governance in which government actors and interested stakeholders from different jurisdictions and organizations came together to address the complex interdependencies emerging at the scale of a specific resource dilemma (e.g., the decimation of endangered wildlife breeding habitat) and across functional areas (e.g., conserved lands, urban forests, and agriculture; Wondolleck and Yaffee 2000; Mullner, Hubert, and Wesche 2001; Ansell and Gash 2008).

Through consensus‐building at formal meetings, all participants engaged directly in the decision‐making process to manage the problem (Ansell and Gash 2008). As an appointed co‐chair of the research and technology development sub‐committee, I facilitated a public consensus‐building process to identify research priorities towards a better understanding of ways to mitigate FD–ISHB.

**FIGURE 1:**
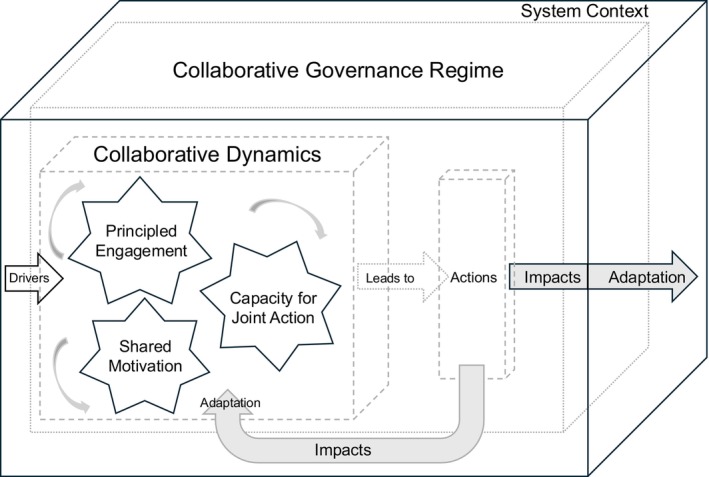
A model of collaborative governance adapted from Emerson, Nabatchi, and Balogh (2012).

In this paper, I conduct an empirical study of collaborative governance in action using the statewide collective action effort that prioritized responses to FD–ISHB. My objective is to examine and contextualize the factors and that led to the successful outcomes of the consensus‐building process. After describing the FD–ISHB problem in further detail, I first review the literature on collaborative governance and identify elements that might lead to different outcomes of the process. Through participant observation and analyses of other cases of governance involving invasive species, the collaborative governance literature, and CISAC meeting materials, I evaluate how the features of this case study apply to other invasive species cases within a contingency model of collaborative governance developed by Emerson, Nabatchi, and Balogh (2012) (Figure 1; see below). I conclude with a discussion of how collaborative governance can be useful in responding to novel plant pathogen threats and how an examination of this case study contributes to the collaborative governance literature more broadly.

### 1.1 Pest–Pathogen Complex– A Complex Management Problem

The avocado industry and land managers of native and urban forest communities in southern California together face the threat of an emergent pest‐disease complex: Fusarium dieback–invasive shothole borers (FD–ISHB; Mendel et al. 2012; Eskalen et al. 2013). The dieback is caused by the combined effects of two ambrosia beetle species from Southeast Asia (the polyphagous and Kuroshio shothole borers; *Euwallacea fornicatus* and *E. kuroshio*), and the specific fungal pathogens each beetle carries (*Fusarium euwallaceae* and *F. kuroshium*; Freeman et al. 2013; Lynch et al. 2016; Gomez et al. 2018; Na et al. 2018; Smith et al. 2019). Over 77 tree species support reproduction of the beetles and their fungi, including avocado, 17 tree species native to California, and ornamental tree species that represent over 25% of all tree individuals planted along streets of southern California (Eskalen et al. 2013; https://ucanr.edu/sites/pshb/Map). As such, the FD‐ISHB pest‐pathogen complex produces devastating impacts at various social‐ecological scales (Eskalen et al. 2013; Lynch et al. 2025). We continue to confirm regular new infestations in many native riparian, oak woodland, and mixed evergreen forest communities, urban forests, and the main avocado‐growing regions of southern California.

In 2003, a single polyphagous shothole borer (PSHB) beetle was caught in a CDFA trap in Long Beach, California. After that, the beetle species went unnoticed until 2012 when it was found damaging backyard avocado and urban forest trees in the Los Angeles basin. A rapid monitoring response uncovered the broad host range of the pest‐disease complex, but its ability to establish in native vegetation was only gradually recognized (Eskalen et al. 2013; Lynch, Eskalen, and Gilbert 2016). In 2014, a separate introduction of Kuroshio shothole borer (KSHB) was detected in commercial avocado groves and green spaces of San Diego County. While spreading throughout commercial avocado groves and urban forests, the magnitude of the problem escalated in 2015 after the beetle‐pathogen complex killed an unprecedented number of native willow trees ( *Salix lasiolepis* and *S. gooddingii* ) in the Tijuana River Valley in San Diego County (Boland 2016). The event quickly prompted local, county, and state land managers and organizations to coordinate and confront the issue. Individual efforts were implemented and loosely coordinated among entities across San Diego, Orange, Ventura, and Los Angeles counties (e.g., Greer et al. 2018). Out of these initial efforts emerged the recognition of a need for a cohesive statewide strategy to address the full scope of the problem across different scales. What followed was a lobbying effort by key natural resource advisors to influence the California state assembly to develop legislation that would provide resources to support a statewide effort to control the spread of the beetle and pathogen to new counties and to prevent further economic losses and damage to landscapes.

### 1.2 California Invasive Species Council (CISAC)

Formed in 2009 by state agencies, the Invasive Species Council of California (ISCC) is an interagency council chaired by the Secretary of the California Department of Food and Agriculture and vice‐chaired by the Secretary of the National Resources Agency (http://www.iscc.ca.gov/). Following established by‐laws (ISCC By‐Laws), the council is the “highest level of leadership and authority in state government” that helps coordinate and facilitate activities aimed at mitigating invasive species impacts in California (http://www.iscc.ca.gov/). The California Invasive Species Advisory Committee Charter (2011) was approved by ISCC to create a committee to advise the council on best measures to forestall the ecological and economic harm caused by invasive species “…based on input from and cooperation with other stakeholders and existing organizations.” Appointed CISAC members represent the scope of knowledge necessary to address the complex issues concerning invasive species (e.g., biologists, industry representatives, regulators, economists, educators, native people, county agricultural commissioners, researchers, public relations specialists) to help advise members of the ISCC.

In January of 2018 the California Invasive Species Council (CISAC) convened a statewide summit that initiated the regional collective action process involving collaboration between stakeholder groups, policymakers, and researchers. Out of the summit came suggestions that were incorporated into Assembly Bill No. 2470, which was co‐authored by Assembly Members Lorena Gonzalez Fletcher (80th Assembly District) and Timothy Grayson (14th District). The Bill allocated $5 million for the execution of a statewide FD–ISHB control strategy and mandated that CISAC build consensus on best measures and funding priorities in cooperation with other stakeholders and existing organizations.

### 1.3 Collaborative Governance Applied to the FD–ISHB Case Study

The consensus building mandate to prioritize FD–ISHB control measures fits into a collaborative governance framework because the pest‐pathogen complex spreads through many different land‐use jurisdictions and involves a complex social network (Table 1), as seen with other transboundary environmental problems such as water pollution or habitat degradation (Bryson, Crosby, and Stone 2006; Kettl 2006; McGuire 2006; Sandström and Carlsson 2008). No single actor in this network is able to develop a comprehensive management plan that will adequately mitigate the threat. The avocado industry in California is governed by the California Avocado Commission, but urban and wildland forests are managed by a conglomerate of stakeholders representing public and private entities. Individual actors thus represent public agency managers, corporations, nonprofits, and policymakers across scales and levels of authority and share similar backgrounds in biology, agronomy, ecology, and resource management as well as a shared concern and vested interest in controlling the FD–ISHB problem.

**TABLE 1:** Stakeholder actors who share an interest or stake in a statewide management strategy to control the Fusarium dieback–invasive shothole borers pest–pathogen complex.

	Organization
International	Comisión Nacional Forestal México (CONAFOR)
Academic	CSU Sacramento
	UC Davis
	UC Riverside
	UC Santa Barbara
	UC Santa Cruz
	University of California Cooperative Extension
Federal	United States Department of Agriculture (USDA)
	Forest Service: Fire
	Forest Service: Forest Health Protection (USFS‐FHP)
	Plant Protection and Quarantine (USDA‐PPQ)
	United States Fish and Wildlife Service (USFWS)
State	California Agricultural Commissioner
	California Board of Forestry and Fire Protection
	California Department of Fish and Wildlife (CDFW)
	California Department of Food and Agriculture (CDFA)
	California Department of Forestry and Fire Protection (CAL FIRE)
	California Department of Resources Recycling and Recovery (CalRecycle)
	State Parks
County	Contra Costa Agricultural Commissioner
	Imperial County Agricultural Commissioner
	Local Enforcement Agencies (LEA)^a^
	Los Angeles County Agricultural Commissioner
	Los Angeles County Botanist
	Orange County Agricultural Commissioner
	Orange County Public Works
	Orange County Waste and Recycling
	San Diego County Agricultural Commissioner
	San Diego County Parks and Recreation
	San Diego County Plant Pathologist
	Santa Barbara County Agricultural Commissioner
	Ventura County Agricultural Commissioner
City	City of San Diego
	Parks and Recreation
	Pest Control Advisor
	Storm Water Division
	San Diego Association of Governments
Non‐Profit	Audubon Starr Ranch Sanctuary
	California Association of Resource Conservation District
	San Diego County
	Santa Monica Mountains
	Ventura County
	Center for Invasive Species
	Irvine Ranch Conservancy
	Ojai Valley Land Conservancy
	Southwest Wetlands Interpretive Association
	The Nature Conservancy
	Western Chapter International Society of Arboriculture
	Wildlands Conservancy
Private	Alliance Care Landscaping Company
	Arborjet
	Davey Resource Group
	Disney
	Dudek Environmental
	ICF International
	Private Arborist
	West Coast Arborists

^a^ Certificated by CalRecycle to ensure the correct operation and closure of solid waste facilities in the state and guarantee the proper storage and transportation of solid wastes.

To assess how collaborative governance can be effective in slowing the spread of FD–ISHB and invasive species more broadly, it is important to understand the contextual conditions likely to facilitate or discourage desired outcomes of collaborations.

## 2 Collaborative Governance Literature Review

The notion of collaborative governance arises from Ostrom's (1990) theoretical and empirical work that challenges Hardin's (1968) position that individuals using a common resource pool will overuse the commons and become trapped and unable to extricate themselves from the problem. Ostrom shows that without top‐down regulation, many are still able to agree on a shared set of rules and avoid this “tragedy of the commons.” Through multiple governing authorities at different scales (i.e., polycentric governance), problems with both local and regional effects can be addressed cooperatively and produce globally positive externalities (Ostrom 2010). Collaborative governance is used interchangeably with other terms relating to environmental management such as network governance, participatory management, and adaptive co‐management (Ansell and Gash 2008; Lubell, Jasny, and Hastings 2017; Nourani, Marianne, and Decker 2018). Here, I adopt the term collaborative governance as the broader theoretical framework employed across many disciplines shows the following: “collaborative” because it indicates a deliberative and consensus‐directed process and “governance” because it includes all aspects of the governing process including management, planning, and policy‐making (Ansell and Gash 2008).

Governance is distinct from management, whereas management refers to everyday decision making and practices (e.g., prescribed burns, tree pruning, vegetation rehabilitation), governance refers to the decision‐making structures, mechanisms, and systems of administration which influence the operation of management systems (Short and Winter 1999). Ansell and Gash (2008) define collaborative governance with an emphasis on six criteria: (1) the forum is initiated by public agencies, (2) participants in the forum include non‐state actors, (3) participants engage directly in decision making and are not merely ‘consulted’ by public agencies, (4) the forum is formally organized and meets collectively, (5) the forum aims to make decisions by consensus, and (6) the focus of collaboration is on public policy or public management. Because this approach has been applied and studied across a range of policy contexts, Emerson, Nabatchi, and Balogh (2012) define collaborative governance more broadly as “the processes and structures of public policy decision making and management that engage people constructively across the boundaries of public agencies, level of government, and/or the public, private and civic spheres in order to carry out a public purpose that could not otherwise be accomplished.”

Collaborative governance models in environmental management have mostly been applied in cases of common pool resources (e.g., fisheries, forest, water; Gerlak, Heikkila, and Lubell 2012). These cases primarily concern issues surrounding resource utilization—how resources are or are not utilized and who decides. By contrast, invasive species management involves issues concerning how common resources are affected by a “common enemy.” In those cases, a common enemy should drive stakeholders to work together because they have a shared vision for what they would like to achieve through collaboration. In reality, however, management of invasive species can be highly controversial because what constitutes a “common enemy” is hotly contested (Crowley, Hinchliffe, and McDonald 2017).

Collaborative governance primarily emerged out of a need to address the rising number and intensity of conflicts over environmental transboundary challenges that could not be effectively addressed through traditional top‐down policy solutions (Gerlak, Heikkila, and Lubell 2012). Proponents of collaborative governance argue that collective action is easier to implement and is more durable than regulation because it enhances social capital, social learning, cooperation, policy learning, and innovation and contributes to democratic principles through transparency and inclusivity (Leach and Sabatier 2005; Bodin 2017). These benefits collectively lead to improved decision‐making, sustained policy implementation, and a better ability to deal with change and uncertainty than a more centralized, rigid bureaucracy (Gerlak, Heikkila, and Lubell 2012).

However, there are as many examples of failures in collaborative efforts as there are successes, highlighting the need for caution in viewing collaborative governance as a panacea for environmental problems (Huxham 2003; Johnson et al. 2003; Bryson, Crosby, and Stone 2006; Ostrom 2007; Ansell and Gash 2008; Muñoz‐Erickson et al. 2010; Bodin 2017). Scholars focusing on collaborative governance have identified key conditions that support or impede successful outcomes of the process.

### 2.1 Collaborative Governance Models

In general, environmental governance should be effective, equitable, responsive, and robust across all elements of governance (i.e., institutions, structures, and processes; Bennett and Satterfield 2018). A number of scholars have interrogated the case study literature in an effort to find a common language for conceptualizing and analyzing collaborative governance in a variety of contexts (e.g., early childhood education policy, green infrastructure development, natural resource management, law enforcement, child and family service delivery, and community planning). Huxham (2003) identified five themes that create pain and reward in collaborative situations: (1) common aims, (2) power, (3) trust, (4) membership structures, and (5) leadership. These themes were then incorporated into more comprehensive collaborative governance frameworks developed through analysis of hundreds of case studies by Bryson, Crosby, and Stone (2006), Ansell and Gash (2008), and Emerson, Nabatchi, and Balogh (2012). None of these frameworks, however, incorporate cases surrounding invasive species management.

The comprehensive collaborative governance frameworks are structured by a set of internal and external factors that influence the process in which stakeholders act collaboratively to make and implement decisions. These frameworks suggest causal pathways among different configurations of those key components. Thus, successful outcomes of the collaborative process in all frameworks are contingent on those key internal attributes of the process itself and external factors that influence the process. Although there are some differences in the ways some elements are configured, there is considerable overlap in how those elements are characterized. The most significant difference is that rather than portraying the outcomes/actions as the endpoint of a linear process, Emerson, Nabatchi, and Balogh (2012) depict those dimensions as influencers that feed back into collaboration dynamics as actions are adapted and adjusted iteratively through more collaborative processes. I adopt the theoretical framework developed by Emerson, Nabatchi, and Balogh (2012) as a basis for analysis of collaborative governance in the context of invasive species management (Figure 1).

### 2.2 Elements in the Collaborative Governance Model (Theoretical Framework)

There is general agreement in the literature about which elements are most important to successful collaborations. The model in Emerson, Nabatchi, and Balogh (2012) is a set of three nested dimensions representing *collaboration dynamics* and *collaborative actions* that are grouped within the *collaborative governance regime* (CGR), which itself is nested within the general *system context* (Figure 1). The system context includes available resources and the policy and legal factors that create opportunities or constraints on processes (Emerson, Nabatchi, and Balogh 2012) and the role of prior relationships or existing networks (Ansell and Gash 2008; Guerrero et al. 2015; Bodin 2017; Tollefson, Zito, and Gale 2012). The drivers that initiate collaboration emerge from this context, which is characterized by the socio‐ecological and historic preconditions that influence the prevailing mode of cross‐boundary collaborative decision‐making (Bryson, Crosby, and Stone 2006; Ansell and Gash 2008; Emerson, Nabatchi, and Balogh 2012). *Collaboration dynamics* are initiated by certain *drivers* and refer to three interacting components that work together iteratively to produce *collaborative actions*: *principled engagement*, *shared motivation*, and *capacity for joint action*. Drivers are the motivating forces that convene participants and set collaboration dynamics in motion; *leadership* and *consequential incentives* are two key drivers present in all collaborative governance models. *Leadership* is considered to be a pervasive influencer within collaborative governance because it can be a “significant outgrowth of collaboration” (Emerson, Nabatchi, and Balogh 2012). *Collaborative actions* lead to *outcomes* and are “the steps taken in order to implement the shared purpose of the CGR” (Emerson, Nabatchi, and Balogh 2012). Each of the components within *collaboration dynamics* consists of their own specific, self‐reinforcing elements. Finally, the cooperative activities achieved through principled engagement and resulting shared motivational benefits help to strengthen knowledge, abilities, skills, resources, and group agency, which also improve institutional structures and processes. This new *capacity for joint action* is the potential that empowers collaborative partners to take effective action towards achieving goals in ways that did not exist before, which further bolsters principled engagement and shared motivation, which reinforces or builds new capacity.

### 2.3 Study Goals

Drawing on a theoretical framework for collaborative governance explained by the elements in the collaborative governance model (Figure 1), I explore how it applies to understanding the conditions in cooperative decision‐making that led to a consensus on statewide priorities to control FD–ISHB in California. My purpose in this research is to (1) use a collaborative governance framework to better understand the CISAC‐stakeholder consensus building process and (2) use that case study to provide theoretical contributions on collaborative governance in the context of invasive species management. Many studies on governance in invasive species management focus on the influence of collaborative network structures on decision making (e.g., McAllister et al. 2015; Lubell, Jasny, and Hastings 2017; Nourani, Marianne, and Decker 2018) rather than on the collaborative processes within those networks. Here, I explore how qualities of the system context, drivers for collaboration, and collaboration dynamics within the collaborative governance regime work together in this case to produce otherwise unattainable actions and forecast how those actions might lead to long‐term outcomes (impacts and adaptation). I further explore whether new themes emerge from the process that promote an understanding of collaborative governance more broadly.

## 3 Methods

Drawing from existing theory on collaborative governance, this research was carried out using qualitative methods, through a combination of participant observation and an extensive review of reports, documents, and case study literature, to understand how conditions during consensus‐building influence process outcomes on a regional scale to control an emergent pest‐pathogen (Stake 1995; Marshall and Rossman 2006; Bernard 2011; Creswell and Creswell 2017; Yin 2017). The overall approach lends itself to an in‐depth exploratory analysis embedded with rich and nuanced detail to illustrate broad general themes and informed insights from participants engaged in collective decision‐making. Participant observation is a qualitative method with roots in ethnographic research in which “theoretical insights are derived from naturally occurring data rather than through interviews or questionnaires” (Huxham 2003). This approach enabled an analysis of group interactions by examining the “how” and the “what” of members' exchanges. Analysis of documents and meeting minutes helped to establish a link between consensus decisions and process outcomes.

Informed participants in the collaboration represented a broad range of perspectives of individuals directly or indirectly concerned about plant health emergencies. They represented entities from county, state, and federal agencies; academic institutions; environmental organizations; state divisions; and private companies (Tables 1 and 2). For consensus building, each of the four sub‐committees (Greenwaste and Firewood as Pathways; Research and Technology Development; Survey, Detection and Rapid Response; Outreach and Education) held public meetings four times at two‐week intervals in March–May 2019, while taking actions between meetings to make progress. As a member of the social community associated with the case, my role as co‐chair of the research sub‐committee presented a unique opportunity to document the case in real‐time as an active participant of the process. My first‐hand involvement in all sub‐committee and most working‐group meetings (see below) naturally placed me in a variety of roles: facilitator, listener, learner, coordinator, science advisor, and fact‐gatherer. As such, this analysis benefits from an in‐depth engagement with stakeholders and deeper understanding of the dynamics and general relationships among them.

**TABLE 2:** Executive and sub‐committee chairs who facilitated collaborative decision making in the present study.

Committee Chair(s)	Title	Affiliation	Code
Executive			
David Pegos	ISCC Agency Liaison; CISAC Executive Director; Special Assistant, Plant Health Division, CDFA	ISCC, CDFA	F
Andy Cline	Entomologist	CDFA	E1
Joe Scheele	Automated Commercial Environment Agent	Department Homeland Security Customs and Border Protection	E2
John kabashima	Environmental Horticulture Advisor, Emeritus	UC ANR–UC Cooperative Extension	E3
Kyle Beucke	Primary State Entomologist/Environmental Scientist	CDFA	E4
Sheri Smith	Regional Entomologist	USDA Forest Service Forest Health Protection (FHP)	E5
Subcommittees			
Research and Technology Development			
Stacy Hishinuma	Forest Entomologist	USDA Forest Service, FHP	RC1
Shannon Lynch	Ph.D. Candidate	UC Santa Cruz	RC2
Survey, detection, and rapid response			
Andrea Hefty	Forest Entomologist	USDA Forest Service, FHP	SC1
Ed Williams	Agriculture Commissioner	Ventura County	SC2
Greenwaste and firewood as pathways			
Thomas smith	Forest Pest Management Specialist	CAL FIRE	PC1
Kevin Turner	Southern California Invasive Pest Coordinator	CAL FIRE	PC2
Outreach and education			
Beatriz Nobua‐Behrmann	Urban Forestry and Natural Resources Advisor	UC ANR–UC Cooperative Extension	OC1

Meetings were conducted via a public online GoToWebinar forum (https://www.gotomeeting.com) and the agendas for each meeting providing access information were distributed publicly in several ways: (1) a permanent list of meetings hosted by CISAC on their website: http://www.iscc.ca.gov/cisac_meetings.html; (2) a collaborative tools information sharing system hosted by University of California Agriculture and Natural Resources: http://anrcs.ucanr.edu/Base‐New/Information_Technology/Web_Development/tools/ctools/; and (3) email notification to roughly 150 actors explicitly requesting they spread the information widely. People were also invited to sign up to receive notices of all the meetings at https://www.cdfa.ca.gov/subscriptions. All public meetings were hosted at the California Department of Food and Agriculture (CDFA) headquarters in Sacramento and recorded using the GoToWebinar system for public use. A designated note taker at each meeting distributed the minutes to the sub‐committee chairs to send to participants for review and commentary, and the final minutes were approved at the following meeting and then posted on the CDFA and CISAC websites. I documented my observations and personal reflections in field notes after each meeting and reviewed publicly available recordings and meeting minutes.

### 3.1 Application of a Collaborative Governance Model

I used collaborative governance frameworks (e.g., Figure 1) as a starting point to identify the prominent conditions influencing the governance processes within the FD–ISHB case and compare it to other cases of governance in the context of pest management (i.e., Mackenzie and Larson 2010; Zalom et al. 2013; Petersen and Wellstead 2014). Accordingly, I used Nvivo qualitative analysis software (QSR International, v. 1.3.2) to code text from public documents, field notes, and 16 transcribed public recordings that related to those key conditions within the theory of collaborative governance (Bernard 2011). I also used open coding on these text data to uncover potential emergent themes not in the literature, progressively grouped themes, and finally theorized a relationship between these themes (Miles, and Huberman 1994). Codes were attributed to speaker identity (e.g., invited participant, sub‐committee co‐chair, executive committee member, note taker) and affiliation (e.g., state agency, NGO, academia); issues of concern (e.g., firewood movement, knowledge gaps, and identified needs); evidence of prior cooperation or conflict (e.g., explaining previous efforts and sharing learned lessons); engagement activities (e.g., seeking broad participation, sharing knowledge, following up, brainstorming, and delegating); intermediate outcomes (e.g., action item, new opportunity, and new partnership); expressions raised in conversation (e.g., expressing enthusiasm or understanding); nonverbal characteristics in conversation (e.g., intonation, pacing, sighing, and laughing); and patterns of listening (e.g., mirroring, asking questions, summarizing, interrupting, and ignoring).

Finally, I used the content from meeting minutes and the *Invasive shothole Borer (ISHB) Strategic Initiative* final report (Lynch 2019) to establish links between collaboration dynamics and process outcomes. The document was reviewed and vetted by executive committee members and selected participants and is publicly available on the ISCC website (www.iscc.ca.gov/ishb.html) for transparency and accountability to legislators who wrote assembly bill no. 2470. The report, which details the outcomes of our efforts, has been distributed to over 500 stakeholders using the UC ANR collaborative tools system and used by the CDFA to appropriate the $5 million towards FD–ISHB management priorities. The report was also used by other funding sources (e.g., USDA Forest Service, CAL FIRE) to fund other priorities not covered by AB 2470.

### 3.2 Limitations

While this study benefits from the deep working relationships I developed with members of the social network involved, there are some important limitations to the methodology worth mentioning. Participant observation allowed me to capture the nuances associated with social interactions in this case, but my conclusions rely on verbal and non‐verbal communication in participant exchanges. There is a risk that consensus was reached because of “group think,” where members in highly cohesive groups reach a premature consensus because they value “harmony and coherence above critical thought” (Janis 1972). The links I make between collaboration dynamics and process outcomes could have been strengthened through additional methods, such as pre‐ and post‐collaboration surveys or in‐depth interviews, that ask a representative sample of participants direct questions related to enhanced social learning and improved actions as a result of cooperation (Blatner et al. 2001). However, because of my position as an insider and participant/leader, it is uncertain whether such data would be subject to response effects that come from respondents “editing” their answers (Bernard 2011). As such, I chose to proceed using naturally occurring data while recognizing those limitations.

## 4 Results and Discussion

### 4.1 Process Outcomes–Collaborative Actions

In theory, collaborative actions (Figure 1) refer to the steps taken to “… implement the shared purpose of the CGR” (Emerson, Nabatchi, and Balogh 2012). The invasive shothole borer sub‐committee of the California Invasive Species Advisory Committee (CISAC) set out to develop essential components of an evolving statewide FD–ISHB Integrated Pest Management (IPM) program and prioritize the use of $5 million to implement the most critical parts of the plan associated with survey, detection, and rapid response (survey), research and technology development (research), and greenwaste and firewood as pathways (pathways) and outreach and education (Outreach). After collaborating in corresponding sub‐committees to build consensus on priorities and projected budgets for each, participants gathered in a follow‐up meeting to decide on priorities for the plan as a whole. Out of this two‐month process of highly focused, dynamic collaboration, participants came to a consensus on a comprehensive set of action steps (Table 3) and long‐term goals that I argue were enhanced by the process, which was supported by the system context, and could not have been attained by any of the organizations acting alone.

**TABLE 3:** Process outcomes (i.e., collaborative actions) that emerged from sub‐committee collaborations.

Category	Action items	Total support
Research and Technology Development	Fund research on: Biocontrol IPM Efficacy Epidemiology Chipping treatments for greenwaste processing FD–ISHB Economic impacts	$2,057,000 (41%)
Survey, Detection, and Rapid Response	Hire one centralized trapping/visual survey coordinator and five surveyors Partner with CAL FIRE to fund hazard tree removal	$2,074,392 (42%)
Outreach and Education	Hire statewide communications coordinator Develop training program for new target audiences. Fund communication operations Develop Rapid Response Tool‐Kit for high‐risk counties	$690,000 (14%)
Greenwaste and Firewood as Pathways	Formalize UC ANR, County Ag Commissioner, and LEA partnerships Expand relationship, survey, and research capacity	$150,000 (3%)

Collaborative governance theory promises new innovations to solving old problems through an enhanced generation of new knowledge through social learning that produces new knowledge integrated with insights from different knowledge systems (Gerlak, Heikkila, and Lubell 2012; Bodin 2017). However, the direct link between collaboration dynamics and collaborative actions is difficult to document empirically because key actions take place over time while under the influence of an evolving system context (Conley and Moote 2003; Koontz and Thomas 2006). In this study, it was easier to attribute enhanced actions as products of features of the decision‐making process because decisions were made over a short time frame, and action items were implemented quickly after the process was completed. The connections between dynamics and actions are evident in the way the action items had impacts across sub‐committees (Table 3). For example, most of the priorities identified by the pathways sub‐committee were addressed through action items prioritized in the other sub‐committees. Those priorities included conducting studies on greenwaste post‐processing treatments (research); prioritizing greenwaste facilities, firewood stockpiles, and distribution sites in survey efforts (survey); and developing paired online‐field training programs tailored to target audiences who focus on greenwaste (i.e., “land management and greenwaste”) and firewood (i.e., “campground and recreation”; outreach; Lynch 2019). In another example, the outreach subcommittee also recognized the imperative need of developing specific printed materials and trainings to be used as an important component of projects identified as priorities by the survey and pathways sub‐committees (Lynch 2019, p.7) in their summary of priorities. These cohesive process outcomes were born out of effective principled engagement, participants' deep understanding, and appreciation of the system context, and the salient forces of leadership and interdependence baked in throughout the project.

### 4.2 System Context and Prior Histories

#### 4.2.1 Cooperation and Conflict

The system context (Figure 1) strongly influenced the success of the FD–ISHB case. Collaborative governance is more likely to succeed when existing social networks are already in place (Bryson, Crosby, and Stone 2006), but the structure of the social network itself (i.e., cohesive, centralized, and compartmentalized; Guerrero et al. 2015; Bodin 2017), institutional, political, and regulatory arrangements (Tollefson, Zito, and Gale 2012) and prior history of conflict or cooperation among network members also factor into its success (Ansell and Gash 2008). The successful outcomes with minimal conflict in the FD–ISHB case largely resulted from local efforts in Southern California that initiated the development a statewide plan, along with lessons learned from 20 years of cooperation and conflict over other important pest problems and fire in California and North America. Examples of experiences from novel‐pest introductions that participants drew from at various points in different sub‐committee discussions include (1) the goldspotted oak borer beetle ( *Agrilus auroguttatus* ) and the pathogen *Phytophthora ramorum* (the cause of sudden oak death), which are responsible for widespread oak mortality in Southern and Northern California, respectively (Rizzo et al. 2002; Coleman et al. 2011; Lynch et al. 2014); (2) the emerald ash borer beetle ( *Agrilus planipennis* ), which has killed hundreds of millions of ash trees in urban forests and wildlands North America; and (3) native bark beetles (*Dendroctonus* spp., *Ips* spp.), which have killed billions of pine trees across millions of hectares of forest in North America in association with climate change (Nordhaus 2009; Petersen and Wellstead 2014); (4) the Asian citrus psyllid ( *Diaphorina citri* ) and huanglongbing disease (HLB), which have caused massive citrus decline in Florida and then established on citrus in Southern California (Warnert 2012); and (5) the glassy‐winged sharpshooter, which vectors the bacterial pathogen *Xylella fastidiosa*, causing Pierce's disease on hundreds of important crops and ornamentals in California (Varela, Smith, and Phillips 2001).

Most of the participants or the organizations they represent were actively involved in previous efforts to respond to their introductions or were highly familiar with the cases because of their widespread destructive impacts on forests and agriculture. A majority of stakeholders were particularly close to efforts involving goldspotted oak borer and bark beetles because of their history in Southern California, where FD–ISHB is having the greatest impact. The bark beetle case involves an interagency collaborative effort, the Mountain Area Taskforce (MAST), that formed after an unprecedented outbreak killed over 14 million trees across 70,000 ha of the San Bernardino National Forest (Merrill 2003; Petersen and Wellstead 2014). This landscape‐level outbreak in the early 2000s was induced by drought and a legacy of fire suppression, posing a significant fire threat to local communities. Two other key high‐value crop pest cases from Northern California were part of the system context because of the state and federal regulatory agencies involved. These pests include the light brown apple moth (*Epiphyas postvittana*), which threatened strawberry, caneberry, and nursery plants in Monterey, Santa Cruz, and San Mateo counties; and the European grapevine moth (*Lobesia botrana*), which impacted grapevine in Napa and Sonoma counties (Zalom et al. 2013).

Four of the above plant health response cases have been studied to understand which factors contribute to prior histories of conflict (emerald ash borer and light brown apple moth) and cooperation (bark beetle and European grapevine moth) in management decisions (Mackenzie and Larson 2010; Zalom et al. 2013; Petersen and Wellstead 2014). The cases provide insights into how the system context was used and contributed to successful collaboration in the FD–ISHB case, but there are important similarities and differences among them worth mentioning. The European grapevine moth and bark beetle cases involve a “bottom‐up” governance approach, whereas the emerald ash borer and light brown apple moth cases represent a “top‐down” form of governance. Interestingly, the light brown apple moth and European grapevine moth cases involve two Lepidoptera species in the Tortricidae family that were introduced to nearby counties in California, but response measures in the light brown apple moth case provoked ire while the European grapevine moth case was considered to be a model response (Zalom et al. 2013). Most importantly, the cases concerning emerald ash borer, light brown apple moth, and European grapevine moth involve cooperation or conflict between the public and technical and regulatory experts while implementing certain responses to plant health emergencies, whereas the FD–ISHB and bark beetle cases concern cooperation among organizations to address pest management challenges.

Prior history of cooperation over FD–ISHB and goldspotted oak borer was clearly acknowledged in many discussions throughout the consensus‐building process, which contributed to creating essential bonds of shared commitment (Emerson, Nabatchi, and Balogh 2012) and facilitated efficient and effective decision‐making under the given time constraints. One member of the executive committee explained in an outreach meeting the following:

…there's a lot of folks on this call and a lot of folks that aren't on this call that have been doing a *ton* of outreach and education work with regard to goldspotted oak borer, firewood, and shothole borers over the last several years. We've been doing it on a shoestring budget basically and it's been an added job to a lot of plates that are already full. And so, I just want for the record that a lot of work has been done, people have been doing tons and tons of work… I mean we've touched millions of people just through state fairs alone and so… everybody ought to be patting themselves on the back for as far as we have come with already full plates and basically almost a zero budget for this.

This deep commitment to engagement entering into the process is recognized to be an important quality in successful collaborative governance (Ansell and Gash 2008) because it is through these prior relationships and networks that “partners judge trustworthiness of other partners and legitimacy of key stakeholders” (Bryson, Crosby, and Stone 2006, p.46). Meeting minutes from each of the inaugural sub‐committee meetings outlined a substantial exchange of ideas, assigned tasks, and designated working groups to drill down on certain issues (Lynch 2019), signifying meaningful progress. At the same time, the overall mood in those meetings was jovial and filled with many moments of levity and laughter. The notable amount of productivity combined with good humor from the start indicated an established sense of trust in existing working relationships, which was maintained and strengthened as the process unfolded. As such, more time could be devoted to getting down to business instead of “remedial trust‐building” (Ansell and Gash 2008).

#### 4.2.2 Established Capacity for Common Purpose

Particular institutional and political dimensions of governance that proved effective in addressing previous landscape‐level pest problems in California (Petersen and Wellstead 2014) provided a model framework for the ISHB sub‐committee, which in turn supported effective engagement and expedient decision‐making once the process launched. The framework can be traced back to 2000 when the California Forest Pest Council and CAL FIRE formed the California Oak Mortality Task Force to work together on minimizing “the impact and spread of *P. ramorum* on natural, agricultural, and human communities” in Northern California (COMTF Partners 2020). The structure consists of a core executive committee and sub‐committees that reflect a “fluid array of multi‐tiered bodies with overlapping and crosscutting jurisdictions, which are typically organized around specific functional tasks” (Tollefson, Zito, and Gale 2012, p.6). A similar integrated response was materialized two years later with MAST in Southern California, which Petersen and Wellstead (2014) recognized as a “new governance arrangement.” The authors reported that the governance structure enabled MAST to achieve short‐ and long‐term goals in protecting mountain communities from looming catastrophic fire threats created from bark beetle outbreaks, and implementation of the plan was well‐received by the public.

The ISHB sub‐committee's institutional arrangements concerning membership and organizational structure (Tables 1 and 2) emulated previous consensus‐driven coalitions that promoted diverse representation at every level of the decision‐making process and set a precedent for inclusive planning and consultation (Tollefson, Zito, and Gale 2012). The sub‐committees represented key “functional components” of the statewide plan, allowing participants to “drill down into” various issues, solutions, and opportunities relating to a specific area of concern within a relatively short amount of time. Sub‐committee meetings coincided but scheduling times did not overlap to encourage participants to attend all meetings. This overall setup addressed common critical barriers to implementing actions and setting priorities associated with landscape‐level pest problems (Petersen and Wellstead 2014).

However, the institutional arrangements also created a unique opportunity for participants to address emerging issues and knowledge gaps at the intersection of the plan's functional components. For example, previous research determined that chipping and solarizing infested wood can kill 99.9% of the beetles and dramatically reduce their risk of long‐distance dispersal in plant material if chipped to pieces smaller than 5 cm or solarized for at least 6 weeks under ideal conditions (Jones and Paine 2015). Therefore, the need for additional research on greenwaste treatments was not recognized until it was discovered through discussions with experts from CalRecycle in the pathways subcommittee that these treatments are not an option for many greenwaste processors who do not have chippers and are required to move their greenwaste material within 48 h. The institutional arrangements consequently contributed to finding better solutions to control FD–ISHB because they created a mechanism to quickly share this new knowledge from unique voices to the people in a position to prioritize more research on greenwaste processing treatments for the state (i.e., the research subcommittee).

In addition to membership and the organizational structure, the ISHB subcommittee's institutional arrangements embodied some degree of formality similar to those in MAST. Co‐chairs in each sub‐committee e‐mailed and posted pre‐approved agendas at least 1 week before every meeting. The itinerary on those agendas followed a specific, predictable order but was flexible enough for fluid discussions. Participant roles were clearly defined. Goals, expectations, timelines, and tasks were explicitly stated at relevant points in every meeting. Meeting minutes were approved following a specific procedure. This level of formality is regarded as a particularly important design feature in governance structures that are facing plant health emergencies because clear, fair, and transparent procedures bring legitimacy to the process so that stakeholders trust that the deliberation has integrity (Fung and Wright 2001; Imperial 2005; Weech‐Maldonado and Merrill 2000; Ansell and Gash 2008). Because there was no formal agreement binding participants to the effort, process transparency was critical to ensuring stakeholders' confidence in voluntarily committing to the process.

Finally, the institutional arrangements in the ISHB sub‐committee reflected an understanding of factors that contributed to cooperation and conflict in previous cases. As Crowley, Hinchliffe, and McDonald (2017) predicted, governance approaches were the primary causes of consternation in the emerald ash borer and light brown apple moth cases in that management decisions rested with the state and were communicated unidirectionally (Mackenzie and Larson 2010; Zalom et al. 2013). Media analysis, focus groups, and in‐depth interviews with individuals directly involved in the light brown apple moth (controversial) and European grapevine moth (not‐controversial) cases revealed that the biggest difference in the European grapevine moth response was the clear presence of local leadership (e.g., County Agricultural Commissioners and Cooperative Extension) investing early in building strong relationships and support networks with the community (e.g., citizen groups, environmental groups, and agricultural industry groups; Zalom et al. 2013). Although public voices were not part of the planning process in the present study, the inclusion of “on the ground” local leadership (Table 1) and a stand‐alone sub‐committee focusing on outreach and education reflects the inclusive and anticipatory approach adopted in the European grapevine moth case because it established a means for local leadership to discuss information about imminent threats with the public ahead of any decisions. Prior efforts established a robust information sharing system through UC ANR collaborative tools, which served to expedite communication of new knowledge or updates from local leadership to the public. Outreach and education committees were also components in California Oak Mortality Task Force and MAST and provided the apparatus for shared decision‐making, critical early face‐to‐face dialogue, and open, responsive communication between neutral, non‐regulatory parties and different groups. This arrangement “enabled MAST representatives to effectively communicate with the public to generate support for forest management actions that prior to the outbreak would not have found support” and “played an important role in moving objectives forward” (Petersen and Wellstead 2014, p.8). The care put into establishing such a system that promotes a well‐coordinated emergency response was also linked to decreased pesticide use and, overall, more sustainable pest‐management programs (Zalom et al. 2013).

Overall, the system context created the capacity for participants entering into the FD–ISHB decision‐making process to achieve a common purpose. Rather than an outgrowth of principled engagement (Emerson, Nabatchi, and Balogh 2012), this capacity for joint action formed the essential leadership structure, which together enhanced effective engagement once the FD–ISHB decision‐making process mobilized.

### 4.3 Leadership

As expected, leadership was instrumental in promoting the successful outcomes produced by the ISHB subcommittee. Engagement was driven, maintained, and strengthened by key leadership attributes. Environmental Horticulture Advisor John Kabashima from UC Cooperative Extension took the initiative to mobilize the necessary people to bring the FD–ISHB issue to the legislature and secure funding for a cohesive statewide plan. While he propelled the process into action, the leadership structure set the direction and tone for effective engagement, which was enhanced by the quality of leadership as the process unfolded.

#### 4.3.1 Leadership Structure

Given that collaborative governance “requires a commitment to a positive strategy of empowerment and representation” (Ansell and Gash 2008, p.552), perhaps the most important boon that emerged from the system context was a strategic hierarchical leadership structure that distributed power across participants and created opportunities for new leaders to emerge (Table 2). Multiple leadership opportunities and roles that reflect various stakeholders' strengths at different points in the CGR are essential to a successful collaborative governance framework (Bryson, Crosby, and Stone 2006; Emerson, Nabatchi, and Balogh 2012; Reed et al. 2018). The ISHB subcommittee consisted of multiple types of leaders who participated in every discussion. The CISAC executive director, who presided over all ISHB subcommittee meetings, provided strong facilitative, administrative, and network leadership and glued all the sub‐committee activities together. Executive committee members participated in decision‐making and liaised with their respective local, state, and federal entities. Co‐chairs led discussions, delegated activities, and shared the workload to conserve one another's time. This collaborative leadership structure created a network of support, a collegial atmosphere, and an added level of accountability, clarity, and procedural transparency and integrity.

The leadership structure also created more opportunities for participants to volunteer for leadership roles as the planning process unfolded and new needs were identified. Volunteers coordinated actions between meetings through smaller working groups within each sub‐committee, and these working groups reported back accomplishments and recommendations to the broader sub‐committee for discussion and consensus‐building. Empowering participants to be part of the decision‐making gave stakeholders a sense of ownership of the process, strengthening their trust and commitment to the project (Ansell and Gash 2008; Tollefson, Zito, and Gale 2012). Working groups also cultivated new and unique working relationships among diverse stakeholders (e.g., researchers and LEA officers; Disneyland horticulturists, and cooperative extension communication specialists), which generated a collective sense of ownership. This shared theory of action contributed to building new capacity for joint action, which is key to ensuring that collaborative actions are implemented (Emerson, Nabatchi, and Balogh 2012).

#### 4.3.2 Facilitative Leadership

The most common theme that emerged from group interactions was established through critical facilitative leadership—the importance of building and strengthening relationships. In a social network analysis of bottom‐up collaborative environmental governance, Guerrero et al. (2015) found that self‐organized networks would still benefit from some degree of facilitative leadership because social and ecological processes propagate across scales and extend beyond the problem‐solving capacity of self‐organized networks. A precedent for goodwill was set at the start of the planning process because of the prior history of cooperation among different individual groups. However, leadership was crucial in building and setting the tone for an inclusive group rapport to ensure broad and active participation and productive group dynamics (Lasker, Weiss, and Miller 2001).

As a facilitator, the CISAC executive director (F; Table 2) actively worked to align participants in the same direction to achieve a shared goal. Examples include interjecting to ask a participant to define an acronym they used and ensure a common understanding; fielding questions; following up with participants to verify that questions or honestly expressed disagreements were addressed appropriately; redirecting discussions back to the main topic when they began to drift; soliciting input from silent participants; checking in with the note taker to ensure key points were “captured”; summarizing threads of conversation into opportunities, action items, needs, or solutions with the group to find consensus on next steps; and acknowledging participants' contributions. Co‐chairs and working‐group coordinators also embodied this style of leadership, creating a culture of inclusive planning and consultation where participants were regularly told “we need your help” and that their time and energy was “really appreciated.”

Facilitative leadership was particularly important in mitigating conflict by allowing participants to express honest disagreements, validating what was shared, and arriving at a mutual understanding to achieve collaborative actions. The following exchange in the second research subcommittee meeting illustrates those efforts when a participant (P65) raised concerns over creating short‐ and long‐term research categories to prioritize projects:

…I think one of the things that we do wrong with most of these kind of emerging pest things is that we only concentrate on short‐term success. …It's still the fundamental knowledge that we lack of uh, the biology of these things and the interactions that ultimately is going to result in the solution. And uhm, in the beginning, I think the whole emphasis on this uhm, short‐term research for political reasons, it seems to me is, is scientifically not smart.

Here, the research co‐chair (RC1) acknowledges P65's concerns and seeks to clarify goals with the help of the facilitator:

RC1 Yeah, I agree. That is, you know, I think the reason for the delineation between those two types of projects is because uh, we would like to see the funding that comes from CISAC, we would like to see results during the three years that the funding will be doled out. And F, do you want to speak a little bit more about that?

F Well, yeah, just, just to that point, that we have the $5 million dollars. So, we're looking for projects that can be funded with a million dollars in the short‐term uhm, and they can have a three‐year duration to fund those projects. And then simultaneously looking for more long‐term projects…. So, the whole kind of goal of this effort is to have a prioritized list that everyone kind of agrees on. So if you, U.S. Forest Service, or CAL FIRE, or uhm you know, Farm Bill Funding comes up with an extra, uhm you know, X amount of dollars, they can just go right down the list uhm, of priority items, because right now it's in difficult for some funders to go “well, there's so many ideas out there,” they they're looking for a comprehensive list of uhm, that have been vetted through a public process so that everyone's kind of on the same page. So, I hope that helps, P65, to understand the difference between…

P65 I do understand it, and I still think it's not a smart way of going about it.

Still not seeing eye to eye, the facilitator asks P65 for more input rather than aiming to convince P65 to adopt a particular point of view and works to identify points of agreement:

F What would you propose?

P65 So, I think what we really need to know first is okay, what, what can be an ultimate solution for this problem? Can we see spraying insecticides as being a solution?

F So, we're with you on that. There has to be some type of uh, solution.

P65 So, I think we just needed to sort of concentrate, let's say, for instance, you know, should we do a lot of monitoring? You know, I think what we need to know is where the bloody thing is, but it would be nice to spend our effort on trying a solution versus saying, “Hey, you guys have this beetle. What are we gonna do about it? Well, we don't know what to do about it.” And so, you know, I think we need to put all our eggs, doors, whatever in trying to come up with a solution. And, uhm you know, and sometimes it is, not something that can be arranged in one or 2 years.

F We just, we're just faced with a pot of money so that we have to get it out the door. Uhm, you know, we're fine with trying to develop long‐term solutions. It's just trying to figure out what those mechanisms are and if that is the goal of this exercise.

P65 All right, well, let's keep on exercising.

The facilitator followed up on P65's concerns later in the meeting when a research need was identified to potentially use available short‐term funds towards a particular long‐term research project:

F And I guess the second point would be, to kind of P65's point earlier, that P65, do you see value in this type of research versus‐ you were just talking about, you know, trying to develop solutions, right. Isn't this a component that, that, should be part of it?

P65 Oh, definitely. I think it's really important to have these long‐term studies to try to determine what goes on. This, this this kind of work is invaluable. And generally, it's not done because it takes too long. Any papers will come up, but it's really important.

F Okay, thank you.

This frank, open exchange exemplifies how leaders used active listening to facilitate a better group understanding of the importance of how short‐term research fits into long‐term goals, which was not clear to everyone up front. Clarity of aims is essential if “joint working partners are to work together to operationalize policies” (Huxham 2003, p.404). This mutual understanding led to participants ranking that particular research project as a top priority in the final meeting, linking the process to outcomes.

The pivotal role of leadership in inclusive planning was especially clear when prioritizing actions under an omnipresent awareness of time scarcity. As the CISAC executive director put it, “we have some very interested legislators that are watching this process and that want us to move forward as quickly as possible, so we don't really have the luxury of additional time, unfortunately.” This time constraint sometimes created a palpable tension between needing to “move on” and ensuring broad participation but was mitigated by executive leadership.

For example, part of every meeting agenda included introductions at the beginning, when each participant stated their name and affiliation and public comment at the end to solicit additional participant input. Introductions and public comment each typically took 20–30 min because there were many participants. While one co‐chair at an inaugural meeting was wrestling with the sincere desire to proceed with introductions but concern, it would “take a little bit too much time to go through everyone,” the CISAC Executive Director interjected to ensure that each participant had the opportunity to introduce themselves. Similarly, the director stepped in when the end of another inaugural meeting approached before getting to public comment, saying, “Well, we need to go through just briefly and make sure we're hearing from folks. That way, we ensure that they contributed.” Leaders expressed a genuine interest in stakeholders' opinions regardless of how deeply they were involved in FD‐ISHB matters. The director's time and care in acknowledging each participant and seeking broad participation demonstrated to everyone that hearing every voice in the room mattered most—even though it meant that every meeting finished 15–20 min late. All leaders embodied this commitment to transparent, fair, and inclusive processes that executive leadership modeled, which is linked to nurturing trust (Davenport et al. 2007; Leahy and Anderson 2008).

### 4.4 Principled Engagement

The direct antecedents of the ISHB subcommittee planning process set the stage for people with different perspectives, skills, and expertise across institutional, sectoral, and jurisdictional boundaries to deftly build consensus on needs, knowledge gaps, solutions, and action items related to statewide FD–ISHB control priorities. After group introductions, participants naturally stepped through topics following a set of iterative collaborative learning phases (Daniels and Walker 2001), which Emerson et al. (2012, p.11) identify as “four process elements: discovery, definition, deliberation, and determination” within principled engagement (Figure 1). Briefly, *discovery* refers to identifying the scope of the problem or challenge, determining capacity needs, investigating facts, and determining shared interests, concerns, and values (Ozawa 1991; Ehrmann and Stinson 1999). Participants then *define* their purpose, objectives, criteria, concepts, tasks, and expectations through continuous consensus‐building efforts. After *deliberation*, the “thoughtful examination of issues” through “candid and reasoned communication” (Emerson, Nabatchi, and Balogh 2012, 12) and *determinations* (e.g., procedural decisions, action items) is made.

Together with a commitment to inclusive planning and consultation, this principled engagement created an explicit operating rationale to set shared goals fairly, freely share knowledge and resources, and efficiently achieve durable collective courses of action. One participant put it as, “I just wanted to thank everybody. I thought this was a pretty productive discussion and kind of focused everybody in a little bit more on how we can…move the whole process forward.”

#### 4.4.1 Process Element Qualities

The quality of the above process elements observed in the ISHB subcommittee's participant exchanges reflected the group's commitment to a thoughtfully designed and comprehensive statewide action plan. Collaborative governance literature highlights the importance of actively seeking broad participation in bringing legitimacy to the process and producing successful outcomes (Ansell and Gash 2008; Novoa et al. 2018; Reed et al. 2018), a common behavior that emerged from group interactions in all sub‐committee meetings. For example, the ISHB sub‐committee worked to cast a wide net ahead of time and invite as many representative people as possible to the project through various communication channels. Additionally, the initial discovery step in the inaugural subcommittee meetings involved co‐chairs soliciting participants' input on who was missing from the discussion and needed to be recruited—before delving into identifying issues, concerns, and opportunities related to the focus of each sub‐committee. This added step of asking participants up front to be involved in carefully thinking through who needed to be at the table signaled a clear commitment to process transparency and inclusive planning and consultation, which is linked to building trust and a shared commitment to achieving goals (Ansell and Gash 2008; Emerson, Nabatchi, and Balogh 2012). These trust building and social learning approaches are used to deal with conflicts and promote effective stakeholder engagement in invasive species management (Novoa et al. 2016, 2018).

Another reliable sign of effective engagement is the acknowledgment of one another's deliberative contributions (De Vries et al. 2011). Responding directly to a colleague's comment was common throughout the sessions and accompanied by a tone of mutual respect, even when people disagreed. The example from the first outreach meeting below highlights this observation when a participant raised concern after a long discussion over revamping existing websites:

P25 I think determining what to do with the map and the website is important but I hope we will shortly get to active outreach as opposed to passive outreach‐ who are we going to target what, what audiences do we think we need to reach other than the discussion we just had about the greenwaste and the chip and mulch users. Um and I think, and I think the legislature might be more impressed by outreach effort, active outreach effort rather than fixing a website.

OC1 Gotcha.

P72 This is P72, I agree with P25.

The above exchange quickly moved the discussion in a new direction. Participants contributed new ideas such as incorporating FD–ISHB educational materials into K‐12 curriculums, reaching out to and working with Homeowners Associations, creating a social media presence, augmenting citizen science programs, and hiring a statewide outreach and education coordinator. The deliberation culminated in a group consensus to create two working groups that drilled down into the details of hiring an outreach and education coordinator and creating a list of existing and needed target audiences (Lynch 2019). Another example includes an exchange that occurred in the third outreach meeting when the facilitator (F) raised the idea of hosting FD–ISHB educational materials on multiple agency websites:

P68 I strongly disagree with that, F, for one reason. If we put the materials on all three, then we have to update the materials on all three…

F No, you just put a link to it. Negative‐ you just, you just put a link to the materials. So, the material will always be up‐updated from the original owner of the document. And then you just put a link to that information. So that's always updated.

P68 Great. Just wanted to clarify that.

F Sure, yes ma'am, no, I agree. Yeah, that's that's an issue. Yes, no, I would, I was suggesting to just, putting the link to the materials so that when it is updated, they all have the same information.

Acknowledgments also came in the form of giving credit to other participants' previous efforts and how they contributed to advancing next actions, as revealed by one participant in the third survey meeting, who volunteered to help develop example survey and rapid response protocols that could be used in the current efforts:

I just want to acknowledge that I just took a lot of what SC1 put together and just kind of reformatted it and took out the actual details on specific uhm trapping uhm methods…. And I want to thank P46 for uhm, kind of sending along the text for the section on zones with infestation. And …. the quarantine section that's something I really don't know anything about so I just did my best with what I had heard from everyone…. Please feel free to edit, add, subtract, delete, whatever and get it back to me as soon as you can and I'll get it back out to everyone before I leave next week incorporating any comments or suggestions.

This example highlights how participants recognized one another's contributions but also demonstrates the important role of interdependence in collaborations and how the collaborative process itself shapes it—a common theme revealed from the group interactions. Ansell and Gash (2008, p.562) explain that “through dialogue with other stakeholders and through achievement of successful intermediate outcomes, they may come to a new understanding of their relationship.” In addition to giving credit to others as appropriate, the participant explained her contribution while recognizing her limitations and the need for more input from other, more knowledgeable group members.

Similarly, in many instances, participants who had never before interacted, asked one another question and shared what they knew to arrive at a shared understanding of the scope of a problem and appropriate next steps. In the example below, PC2 starts a discussion in the first pathway meeting over issues concerning how to track greenwaste materials. A participant from CalRecycle (P12), who was an expert on all the greenwaste facilities in the state but did not know about the current distribution of shothole borer around those sites, wanted to understand previous surveying efforts better:

P12 Uhm, PC2, do we actually have some trapping that was done that shows, this is P12 from CalRecycle, that shows, you know, shothole borer near sites? Is what I'm hearing?

PC2 I'm going to defer to some of the folks in the audience. I know there was a site down in Orange County uh where they had that occur and I believe that is also true in some other counties. Is there somebody? E3? OC1? That can speak to that or RC2?

P67 This is P67. We actually trapped around a number of greenwaste facilities in Los Angeles County and detected shothole borers within about 100 to 200 m of each locations. That was in 2017.

P12 What kind of facilities were they? Do you know?

P67 Um, they were bio‐waste facilities where landscapers would bring all kinds of greenwaste and they chipped on site and then they went either from, the material was either then sold back to landscapers to use as mulch or it was sent to a bioenergy facility.

P12 Okay.

PC2 So I believe there is an opportunity or a need to, perhaps, there's the survey, the uh detection and rapid response folks that maybe will address putting out traps around greenwaste sites and whatnot. But it is an issue…

In sharing their knowledge and experience with one another, it became clear to the group of the need to monitor greenwaste facilities to understand better the role of greenwaste in FD–ISHB long‐distance dispersal. This shared perspective created cohesiveness among those involved, a shared understanding of the problems they collectively faced, and, most importantly, the ability to implement the necessary solutions using the proper mechanisms. Bringing these entities together in the collaborative process opened the door to creating new partnerships between local county agricultural commissioners and local enforcement agencies (LEAs), who previously did not cross paths (Table 3). Similar to the relationships between CDFA and local county agricultural commissioners, who were charged with implementing a trap monitoring program, CalRecycle delegates enforcement authority to local enforcement agencies (LEAs), who have established trusting working relationships with greenwaste processors. Because agricultural commissioners did not have a history of working with greenwaste processors, the partnership with LEAs was imperative to facilitate communication between them so they could access their sites and deploy monitoring traps.

In sum, these exchanges demonstrate how the process of principled engagement and a commitment to inclusive planning and consultation allowed the ISHB sub‐committee to leverage knowledge from a range of perspectives and augmented capacity for joint action. Engagement also enhanced group learning, trust, and interdependence, creating the social capital that motivated participants to work together to develop unique and comprehensive collaborative actions.

#### 4.4.2 Contributing to the System Context

In this study, the system context influenced collaborative processes in a positive and meaningful way. Participants recognized this but, even more, advocated for outcomes that feedback into and improve the system context to better equip future stakeholders in responding to “the next big thing.” The following statement from the pathways co‐chair in a survey sub‐committee meeting provides a useful example:

I just want to say that part of this rapid response, idea of rapid response is trying to identify key players, agencies, and other groups before the infestations even arrive so you're ready to come up with a rapid response plan. Also, identify issues like where would funding come from to help private property owners, etc. And just a couple examples with goldspotted borer in Riverside County. There had been the Mountain Area Safety Taskforce created because of bark beetle kill back in the early 2000s. And when goldspotted borer showed up, they they already had all the agency in there working together– Caltrans, the fire agencies, forest agencies, the public utility companies, and whatnot. They were already used to working together on the fire issue, they immediately turned around and were able to take action on goldspotted borers. So, having that kind of organization up, kind of figured out up front before it actually, the pest that actually arrives can be very valuable.

The statement was essentially a call to participants to put systems in place that elicit an effective response to new FD–ISHB introductions but to also consider that those efforts will have benefits beyond the current system, similar to how MAST efforts benefited the goldspotted oak borer response. Thinking more broadly was encouraged in many instances. Another example includes a discussion over a statewide outreach and education coordinator position as working group members reported their efforts back to the outreach sub‐committee:

P31 …one thing that P69 and I discussed was including room and for other emerging tree pests. You know, do we want to coordinate any new messaging with our shothole borer messaging? So in the beginning, I think, she changed the title of the position a little bit.

P69 Yeah, one, one thing that I wanted to add is going even beyond the position itself. I just strongly encourage this committee to really do some long‐term thinking when we do things like establish those social media presence and make sure that are developing something that is sharing a message that this is not just this one pest and when it you know, if…we, you know, solve this problem, the whole concept doesn't go away.… so when you even then, like your name on Facebook page, Twitter account or something like that, that we don't sort of pigeonhole too much just into shothole borer.

P40 Yes, thanks. I just want to first thank P69 for those ideas. I think it's, it's wonderful. And it'll be actually a savings in the long‐term to the state and coordination of addressing invasive pests, because it's, what she's suggesting, creates a template…that can be used in and made specific to each species. So thanks, P69.

In a pathways sub‐committee meeting, the *Don't Move Firewood* national campaign manager from The Nature Conservancy raised a similar point while discussing firewood pest treatments, saying “And when you guys talk about these issues in general…I would urge you to not focus on the shothole borers biology in driving your treatments. In case another pest rears its ugly head that has a more durable biology.” Similarly, the executive committee member representing the U.S. Forest Service communicated to the research sub‐committee that “APHIS is looking for some consensus here locally on what some of the Research and Technology Development needs they might fund for farm bill proposals at the national level.” Communicating this message had the added benefit of incentivizing participants to work together because their efforts had long‐term advantages by creating new opportunities at the national level.

Finally, a sincere appreciation of the system context and who collaborative decisions impact was revealed in discussions concerning management activities and how to ensure good working relationships with the public. These considerations were particularly clear in discussions over rapid response activities that potentially involve removing high risk, newly infested trees from private properties. In the following example from a survey meeting, the CISAC executive director (F) consulted with survey and pathways sub‐committee co‐chairs (SC2 and PC1‐2; from the Ventura County Agricultural Commissioner's Office and CAL FIRE respectively) over the issue:

I mean the only issue is, say you have a heavily infested tree, without homeowner permission to remove the tree what do we do? Under that scenario and the homeowner says “no, I don't want the tree removed.” Um, what's the scenario? How does that play out I guess, I'm just curious?

After some discussion over who has the authority to remove trees on private property (e.g., CAL FIRE, versus County Agricultural Commissioners) and how the regulatory process works, the group discussed alternative approaches:

PC2s …. I'd like to suggest on the uh tree removal maybe at this stage in the game we should just go with voluntary participation by private property owners at least to get the property to the program off the ground. It may be in year two or three try and go in and take trees if people aren't willing.

SC2 Okay, I think that's a reasonable approach.

F SC2, I just wanted to add a little color to that conversation that we've been very successful working with um, citrus tree owners who refused to remove their trees. We do have the authority to remove their trees. However, we try not to use that and so will triangulate and just sic a bunch of different experts on them. You know, we'll start with our staff or, you know, a master gardener or the county Ag Commissioner or depending on kind of where their issue is you got to figure out the person and it's been really helpful and kind of triangulating and making that person understand that there is a reservoir for the disease and so, y'u need to remove it. And it usually takes multiple tries but we've been pretty successful…

SC2 No I agree with you F. Uhm, I I think that most homeowners if given the information that the tree is likely to die and is likely to become a hazard and is likely to become a fire hazard at some point will probably agree to allow the removal of the tree. But I think the biggest problem is is whether they end up paying for that removal or whether um, whether if some of the funds that are available can be used to remove those trees.…And probably the best entity to do, to do those removals would be professional uh tree companies, arborists.

This exchange highlights a key similarity between FD–ISHB and the European grapevine moth case, which was considered a model emergency response, and the light brown apple moth and emerald ash borer cases, which resulted in law‐suits, public outrage, and a loss of institutional trust (Mackenzie and Larson 2010; Zalom et al. 2013). Moreover, public pressure resulted in the early termination of light brown apple moth treatment activities. Discussions like the example above led to action items for the outreach sub‐committee to develop mechanisms for neutral, independent, non‐regulatory parties to engage in face‐to‐face dialogue with the public—before there is even a problem. Interestingly, this strategy was adopted in the European grapevine moth emergency response. Interview respondents involved in both light brown apple moth and European grapevine moth responses “expressed a sense that if the process they had experienced had been used at the onset of the light brown apple moth emergency that the ultimate outcome would have been different” (Zalom et al. 2013, p.v). All the examples above demonstrate how participants of the ISHB sub‐committee carefully thought through how the outcomes of their current efforts will impact the system context and, more importantly, how to ensure long‐lasting beneficial outcomes.

## 5 Conclusions

It is no surprise that responses to novel landscape‐level pest introductions can be controversial. Making decisions is not an easy enterprise in the face of an unexpected pest arrival with uncertain social and ecological ramifications. Decision‐making is further entangled when those introductions result in outbreaks that spread across multiple land‐use jurisdictions, rendering any single entity impotent to fully address the scale of the problem. However, the source of friction associated with most pest introduction responses is usually predictable—more often than not, escalated conflicts can be traced back to a top‐down governance approach that was communicated either unidirectionally, with an unhelpful tone, or both. This study highlights how using collaborative governance to control a major pest‐pathogen complex can lead to thorough and productive pest control strategies and effectively mitigate conflict. Analysis of participant observation and public document data confirmed that the comprehensive set of collaborative actions that emerged from a statewide deliberative and consensus‐directed process to control FD–ISHB spread and impacts were due to conditions identified in theoretical frameworks for collaborative governance (i.e., Emerson, Nabatchi, and Balogh 2012). This instance represents a model of the “best‐case” scenario that could be adapted by other pest and invasive species management cases and help decision‐makers prepare for “the next big thing.”

The action steps in this case study were enhanced by the structure and quality of principled‐engagement process elements and could not have been attained by any organization acting alone. However, these processes greatly benefited from established social mechanisms supplied by the system context that helped to establish process transparency and legitimacy entering into the project. Drawing from prior successful cases (i.e., MAST), institutional arrangements were organized into multiple intersecting “functional components” of the plan that were glued together by an executive committee and a facilitative leader: (1) greenwaste and firewood as pathways; (2) research and technology development; (3) survey, detection, and (4) rapid response; and (5) outreach and education. This structure allowed participants to drill down deep into certain focus areas while addressing issues and knowledge gaps at the intersection of the plan's functional components. Additionally, embedding outreach into the plan indicated a commitment to anticipatory engagement with the public and other stakeholders and created the apparatus for critical early face‐to‐face dialogue and shared decision‐making between neutral, non‐regulatory parties and different groups.

The setup also generated a collaborative leadership structure consisting of multiple leadership roles and allowed new leadership to emerge, reflecting a shared sense of ownership of the process and a commitment to a positive strategy of empowerment and representation. As a component of the leadership structure, facilitative leadership was instrumental in mitigating conflict, establishing clear expectations, and aligning participants in the same direction to achieve a shared goal. This well‐established strategy of inclusive planning and consultation created the capacity for participants to achieve a common purpose entering into the FD–ISHB decision‐making process.

A spirit of inclusivity was sustained and strengthened as participants representing different entities engaged in developing new ideas, projects, and partnerships. Members were committed to actively seeing broad participation, and participants' contributions were acknowledged and met with a tone of mutual respect, even when disagreements were expressed. Ultimately, participants in the ISHB sub‐committee devoted their time and energy to a short but intensive planning process resulting in more capacity for joint action, trust, interdependence, and a robust action plan that was quickly implemented.

Essentially, the elements that contributed to productive and rewarding outcomes in this study are consistent with expectations in the literature (Ansell and Gash 2008; Emerson, Nabatchi, and Balogh 2012). Although this particular pest problem is not shrouded in controversy, the collaborative governance pieces that contributed to a rewarding group effort in this case could still be applied to more thorny situations, with some modifications as appropriate. For example, high conflict scenarios might require a professional mediator in place of a facilitator to address differences in views or deep resource and power inequities.

Ultimately, the CISAC FD–ISHB sub‐committee came to a consensus on the allocation of $5 million towards the control of FD–ISHB in California (Table 3). This resulted in five fully funded research projects, two new statewide positions to coordinate trapping and communication activities, two novel partnerships between government agencies and cooperative extension to cover monitoring activities and mitigate greenwaste movement, and a statewide outreach and education plan.

Further research will need to determine whether the collaborative actions implemented in this study result in improved environmental outcomes (Gerlak, Heikkila, and Lubell 2012) or whether the rewards from the statewide FD–ISHB collaborative efforts are ephemeral. Given that the participants in these efforts were deeply committed to the cause, are highly interdependent, and make conscious decisions to incorporate long‐term benefits in short‐term planning, I expect that the outcomes identified in this study launched an effective statewide integrated pest management strategy to control FD–ISHB. I expect that the strategy also provides a useful template that will help prepare stakeholders' responses to future novel pest introductions. Simply put by one participant at the end of these efforts, “I'm getting really excited about this.”

## Conflicts of Interest

The author declares no conflicts of interest.

## Data Availability

The author confirms that the data supporting the findings of this study are available within the article.
